# Psychological Distress, Related Work Attendance, and Productivity Loss in Small-to-Medium Enterprise Owner/Managers

**DOI:** 10.3390/ijerph10105062

**Published:** 2013-10-15

**Authors:** Fiona Cocker, Angela Martin, Jenn Scott, Alison Venn, Kristy Sanderson

**Affiliations:** 1 Menzies Research Institute Tasmania, University of Tasmania, Private Bag 23, Hobart TAS 7000, Australia; E-Mails: Alison.Venn@utas.edu.au (A.V.); Kristy.Sanderson@utas.edu.au (K.S.); 2 School of Management, University of Tasmania, Locked Bag 1316, Launceston TAS 7250, Australia; E-Mail: Angela.Martin@utas.edu.au; 3 School of Psychology, University of Tasmania, Private Bag 30, Hobart TAS 7001, Australia; E-Mail: Jenn.Scott@utas.edu.au

**Keywords:** depression, workplace, absenteeism, presenteeism, productivity, SMEs

## Abstract

Owner/managers of small-to-medium enterprises (SMEs) are an under-researched population in terms of psychological distress and the associated health and economic consequences. Using baseline data from the evaluation of the Business in Mind program, a mental health promotion intervention amongst SME owner/managers, this study investigated: (i) prevalence of high/very high psychological distress, past-month sickness absenteeism and presenteeism days in SME owner/managers; (ii) associated, self-reported lost productivity; and (iii) associations between work, non-work and business-specific factors and work attendance behaviours. In our sample of 217 SME owner/managers 36.8% reported high/very high psychological distress. Of this group 38.7% reported past-month absenteeism, 82.5% reported past-month presenteeism, and those reporting presenteeism were 50% less productive as than usual. Negative binomial regression was used to demonstrate the independent effects of socio-demographic, work-related wellbeing and health-related factors, as well as various individual and business characteristics on continuous measures of absenteeism and presenteeism days. Health-related factors (self-rated health and treatment) were the strongest correlates of higher presenteeism days (*p <* 0.05). Work-related wellbeing factors (job tension and job satisfaction) were the strongest correlates of higher absenteeism days (*p <* 0.05). Higher educational attainment, treatment and neuroticism were also correlated with more absenteeism days. SME-specific information about the occurrence of psychological distress, work attendance behaviour, and the variables that influence these decisions, are needed for the development of guidelines for managing psychological distress within this sector.

## 1. Introduction

Depression is a costly health problem in the labour force [1,2,3,4]. In addition to the health care resources required to treat it, depression impacts workers’ behavioural, cognitive, emotional, interpersonal, and physical functioning, leading to excess disability and sickness absence [5,6], and impaired work ability [7]. Therefore, a large proportion of the cost of depression can be attributed to lost workdays due to absence and the reduced productivity of individuals who continue working when ill (presenteeism) [4]. Recent annual estimates have reached 44 billion dollars in the US [4], 15.1 billion pounds in the UK [8] and 12.6 billion dollars in Australia [9].

### 1.1. Background and Rationale for the Study

Despite the economic impact of depression-related presenteeism, few studies have attempted to identify which work-, non-work-, or health-related factors are associated with continued work attendance amongst workers reporting depression or related psychological distress. Without information regarding which influential factors are amenable to change and/or intervention, employers, business owners and occupational health professionals may struggle to manage this behaviour, and moderate the costs. One sector of the workforce for whom such evidence could prove particularly beneficial is small to medium enterprises (SMEs) which have been largely neglected by occupational health research.

SMEs contribute significantly to continued global economic growth and the generation of new jobs internationally [10,11], making the promotion of organisational health and productivity in this setting is vital [12]. This could be achieved, in part, by improved management of work stress, subsequent poor mental health and related absenteeism and presenteeism. However, there is scant SME-specific literature to inform targeted occupational health interventions. As a result, available resources are often informed by research conducted within larger organizations and developed without evidence that factors associated with poor mental health and associated work attendance behaviours in large organizations are influential within SMEs. This dearth of SME-specific information has limited the development of workplace health promotion and intervention programs tailored of the needs of this sector and may explain why strategies routinely employed by larger organizations, such as mental health literacy workshops, or stress management training, are difficult to implement and infrequently adopted by SMEs. 

Organizational features common in SMEs, such as multiple roles, long work hours and emotional and financial commitment may increase the likelihood of experiencing role ambiguity, work/life imbalance, and financial pressure, all of which have been identified as precipitants of job stress, burnout [12], psychological distress, and depression in larger organizations [13,14,15,16,17]. However, this is yet to be corroborated. Further, exploration of the antecedents of sickness absence has identified organization or firm size as positively correlate of absenteeism [18,19,20,21,22,23,24,25,26,27]. Consequently, the prevalence of presenteeism may be much higher in SMEs as absenteeism behaviour is less likely as a response to mental health issues and reduced coping with work-related stress. Continued work attendance may also be more common in SMEs as small teams reliant on interdependent co-worker productivity prompt individuals to continue working when unwell [19,28]. That is, the small team factor may necessitate the adoption of multiple role responsibilities which make it difficult for co-workers or managers to compensate for the diminished work capacity of a worker who continues to work whilst ill, thus increasing the subsequent cost [20,29]. However, these elucidations are similarly unconfirmed. 

This study aimed to address the aforementioned dearth of SME-specific literature by performing, to our knowledge, the first exploratory analyses of the occurrence and consequences of high/very high psychological distress amongst a sample of SME owner/managers. Firstly, it aimed to identify the proportion of participating SME owner/managers reporting high/very high psychological distress. Secondly, it aimed to identify the prevalence of past-month sickness absenteeism and presenteeism days reported within this sample of SME owner/managers reporting high/very high psychological distress, and the associated, self-reported lost productivity. Finally, this study aimed to identify which work, non-work and SME-specific factors are associated with these work attendance behaviours within this sample of SME owner/managers reporting high/very high psychological distress.

### 1.2. Theoretical Approach

**Figure 1:**
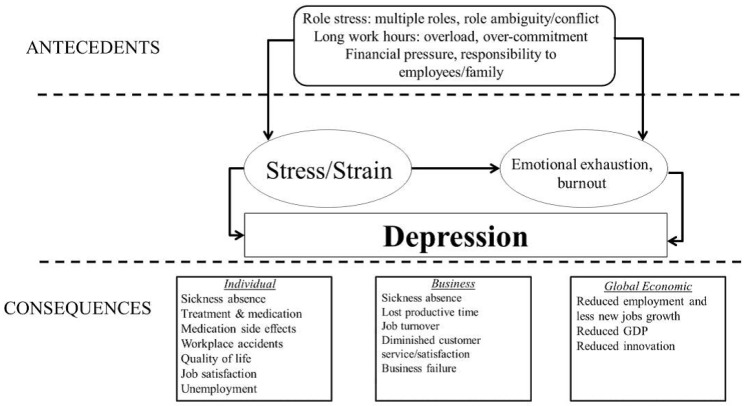
Potential antecedents and consequences of depression in small-to-medium enterprise owner/managers.

The authors developed a model (Figure 1) based in existing literature to conceptualise how various individual and business characteristics, and work and non-work factors may contribute to the development of depression and psychological distress in SMEs, and how they correlate with work attendance decisions [30]. The selection of potential correlates of absenteeism and presenteeism was informed by research which suggested presenteeism should not be examined in isolation, but with reference to the significant knowledge acquired through absenteeism related research [31,32]. Specifically, Aronsson’s [32] theoretical model for research into sickness presenteeism which suggests sickness absenteeism and presenteeism are alternatives of the same decision process, Hansen and Anderson’s [33] model which schematizes the influence of organizational and individual factors on work attendance decisions, and the literature review and the model [34], which suggests certain features of the work context, worker characteristics, and work experiences may influence absenteeism and presenteeism decisions, were used. Therefore, a range of socio-demographic, individual and business characteristics, and work-related wellbeing and health-related factors were selected based on their identified association with absenteeism and/or presenteeism behavior within larger organisations.

## 2. Experimental Section

### 2.1. Participants, Sampling and Recruitment Procedures

As part of a larger study examining the feasibility and efficacy of a workplace mental health promotion program targeted at small-to-medium enterprise (SME) owner/managers, baseline data prior to intervention was available for analysis. To be eligible to participate in the study, owner/managers needed to be in a managerial role within a business employing less than 200 employees, be over 18 years of age and have access to a telephone and computer/DVD player, to be used for delivery of the intervention materials. 

Data in this paper comes from the first 217 consecutive enrollees in the trial. Data were from owners and managers who were aggregated into one group, “owner/managers”. The majority of respondents identified themselves the business owner, CEO or director (67.3%). The remaining 32.7% identified themselves as senior managers, with a high level of responsibility regarding the day-to-day running of the business, and subject to many of the same operational, workplace stresses as the SME owners. Therefore, aggregation is unlikely to influence the results of this study. Whilst differences between entrepreneurs and small business owners in terms of creativity and risk taking propensity are of cited [35,36], recent research suggests there are fewer differences between small business owners and managers, and that small business owners are more comparable to managers than to entrepreneurs [37]. Further, a series of one-way ANOVAs revealed no significant differences between owners and managers reporting high/very high psychological distress on any of the correlates of absenteeism and presenteeism explored in this study or the number or occurrence of sickness absence and presenteeism days.

As we used various, diverse avenues of recruitment the population used in this study should be considered a convenience sample and is in no way structured to be representative of the broader, Australian SME population. It is therefore not surprising that the sample was different to the source population of Australian SMEs in terms of percent female, and industry (Table 1). In comparison to Australian comparative data, our sample was more likely to be female, and from the health industries. 

**Table 1:** Sample characteristics of Business in Mind participants compared to Australian Bureau of Statistics, SME demographic information.

Sample Characteristics	Business in Mind	Australian Bureau of Statistics
*Age*	33.2%—40–49 years	28.2%—45–54 years
	25.8%—50–59 years	26.8%—35–44 years
	23.9%—30–39 years	14.3%—55–59 years
	9.2%—18–29 years	12.1%—25–34 years
	7.8%—60–69 years	<10%—65 and over
		<10%—25 and under
*Gender*	56.7%—female	31.5%—female
*Industry*	29.5%—other	83.1%—service
	20.3%—service	10.2%—agriculture, forestry, fishing
	14.3%—health	4.1%—manufacturing
	6.0%—building & construction	0.4%—mining
	7.8%—retail	
	3.7%—innovation, science, tech.	
	4.2%—finance	
	3.7%—manufacturing	
	3.7%—transport	
	1.4%—agriculture	
	2.8%—tourism	
	2.3%—wholesale	
	0.3%—mining	

### 2.2. Measures

#### 2.2.1. Kessler (K10) Screening Scale for Psychological Distress

The Kessler 10 (K10) Screening Scale for Psychological Distress measured current psychologist distress. This 10-item measure asks about the level of anxiety and depressive symptoms a person may have experienced in the four weeks prior to completing an interview or questionnaire. For example, “In the past four weeks, how often did you feel tired out for no good reason” and “In the past 4 weeks, how often did you feel nervous”. 

Each item is measured on a five-level response scale: none of the time (1); a little of the time (2); some of the time (3); most of the time (4); and all of the time (5). Scores of the ten items are summed, yielding a minimum possible score of 10 and a maximum possible score of 50. Low scores indicate low levels of psychological distress and high scores, high levels of psychological distress. The scores in our study were grouped according to the criteria developed by Andrews and Slade [1] into four levels of psychological distress: low (10–15); moderate (16–21); high (22–29); very high distress (≥30). These cut off scores were used in the 2000 Health and Wellbeing Survey (conducted in Western Australia), and the Australian Bureau of Statistics’ (ABS) 2001 National Health Survey, to estimate levels of psychological distress [38]. For the current analysis, the K10 was dichotomised at low to moderate levels of distress (K10 ≤ 21)*versus* high to very high levels of psychological distress (K10 ≥ 22) [39]. This categorisation was chosen as high to very high levels of psychological distress have been shown to be associated with clinical diagnoses of anxiety and affective disorders [1,2]. 

The K10 was developed based on extensive psychometric analyses, in large general population samples, using modern item response theory methods to maximize the scale’s precision and to ensure each item in the scale had consistent severity across socio-demographic subsamples [40]. It is regularly used in population health surveys to measure psychological distress and has greater discriminatory power in detecting DSM-IV depressive and anxiety disorders than other short general measures, such as the General Health Questionnaire (GHQ-12) [41]. 

#### 2.2.2. Absenteeism and Presenteeism Days and Related Lost Productive Time

Absenteeism days were measured using an item from the World Health Organizations Health and Work Performance Questionnaires (HPQ). Specifically, “In the past 4 weeks, on how many days did you miss a whole day of work because of problems with your physical or mental health?” [42,43]. HPQ validation studies show good concordance between measures of self-reported absenteeism and pay-roll records over a 30-day recall period [43,44]. Further, these types of recall-based questions have been typically used in previous studies to establish absenteeism rates for mental disorders [45]. Using this question also allowed the examination of the correlates of absenteeism amongst owner/managers with high/very high psychological distress as a continuous (number of days) and a dichotomous (none/any) measure.

Our presenteeism measure, like the absenteeism measure, had a 4-week recall period. The first presenteeism measure determined the number of days an owner/managers attended work while suffering from a health problems/s (presenteeism days). This was assessed by the item “How many days in the last 4 weeks did you got to work while suffering from health problems?” [46]. The responses to this item were dichotomised and used as the outcome in our regression analyses. Owner/managers also provided a self-reported estimate of lost productive time associated with their presenteeism days, on a vertical scale from 0%–100%, in answer to the item “On these days, when you went to work suffering from health problems, what percentage of you time were you as productive as usual?”. Therefore the measure of presenteeism days could be adjusted by a percent rating of perceived productivity [47] to estimate lost productivity from being at work when sick [48]. This measure was used to assess the mean lost productive time reported for owner/managers reporting low/moderate psychological distress compared to those reporting high/very high psychological distress. These measures have been validated in population of employed, individuals reporting symptoms of depression and anxiety [49]. The correlates of presenteeism as a continuous (number of presenteeism days) and dichotomous (none/any) measure were examined.

#### 2.2.3. Socio-Demographic Factors

As older workers, females and those with post high school education have been more likely to report presenteeism behaviour [32,33,50], sex (male, female), age and education were included in our analyses. Age was initially grouped as 18–29 years, 30–39 years, 40–49 years, 50–59 yeasts, and 60–69 years and 70+ years. However, due to small numbers, age was collapsed in to three categories (18–39 years, 40–49 years, and 50+ years). Similarly education was reduced from five groups (secondary school, higher school certificate/matriculation, diploma/associate diploma, university degree and other) to two (post high-school, no post high-school). This classification has been used in previously published studies [51].

#### 2.2.4. Individual Characteristics

Conscientiousness was chosen as a potential correlate of absenteeism and presenteeism as previous studies revealed its negative association with absenteeism [52,53,54,55], and continued work attendance has been positively associated with psychological hardiness, conscientiousness, and the inability to refuse the demands of others, known as “individual boundarylessness” [32]. Further, entrepreneurs and small business owners have been shown to score higher on conscientiousness when compared to other professionals [56,57]. Conscientiousness was measured at baseline using a five-item measure from the NEO Personality Inventory-Revised (NEO-PI-R) [58]. The validity and reliability of this measure has been tested in studies of occupational samples [58,59].

#### 2.2.5. Business Characteristics

Business characteristics selected for investigation were whether the owner/managers supervised employees, firm size, and hours worked per week. Firm size was included as a as [18,28] smaller work teams and inter-dependent co-worker productivity may affect work attendance decisions as managers who cannot rely on the back up of multiple employees may continue working when ill to reduce the potential lost productive time [28,60]. Whether or not an owner/manager supervised employees and work hours were also considered and expected to be positively associated with presenteeism. Individuals who work in a supervisory role and/or routinely work more than the average numbers of hours per week are likely to feel under greater time pressure if they take time off for illness [33] as they may feel their work tasks will accumulate in their absence [32]. Additionally, the sense of responsibility that comes with a supervisory role may prompt continued work attendance. Further, team designs are often heavily reliant on task interdependence where one worker’s output is dependent on their colleague’s output. Johns [31] posited that task inter-dependence is likely to be negatively correlated with absenteeism and positively correlated with presenteeism. Such pressures may be magnified in SMEs as managers and employees are dependent on each other as there are fewer people to take on the tasks of a sick colleague. 

Number of employees supervised (“How many employees you directly supervise in your current team”) was categorised as none *vs.* any employees. Number of employees within an organization was measured in response to the “How many employees work in your business/organization (full time equivalent)”. Responses were categorised as no employees, 1–4 employees, 5–19 employees, and 20–199 employees. Hours worked was also included and was determined in response to the item “How many hours, on average, do you work each week?”. Due to relatively small numbers responses were then categorised as 0–40 h and 40 h or more hours.

#### 2.2.6. Work-Related Wellbeing Factors

Work-related wellbeing factors explored were business confidence, job satisfaction, and work/life balance. The inclusion of these factors in this study was prompted by previous research which revealed absenteeism is associated with work overload [61], and low job satisfaction [62], and poor work/life balance is associated with both absenteeism and presenteeism [51,63]. Further, poor work/life balance is a commonly cited by-product of SME management or ownership [64,65,66,67] and Caverley *et al.* (2007) found that job insecurity stemming from downsizing and restructuring exaggerated levels of presenteeism [68]. In this study business confidence was employed as a SME-specific indicator of job security and its association with work attendance decisions was explored. 

Business confidence was measured by owner/managers indicating, on a 6-point scale (strongly disagree, disagree, somewhat disagree, somewhat agree, agree, strongly agree), their level of agreement with the statement “I feel confident about the business’ performance over the next 12-months”. Responses were then categorised into confident (somewhat agree, agree, strongly agree) and not confident (somewhat disagree, disagree, strongly agree. Job satisfaction [69] was assessed by a 3–item measure which required owner/managers to indicate their level of agreement (strongly agree, agree, neither agree nor disagree, disagree, strongly disagree) with the following statements “Overall, I am satisfied with the kind of work I do”, “Overall, I am satisfied with the organisation in which I work” and “Overall, I am satisfied with my job”. Job tension [70] was assessed by a 4-item measure which required owner/managers to indicate their level of agreement (strongly agree, somewhat agree, agree, somewhat disagree, disagree, strongly disagree) with the following statements “My job tends to directly affect my health”, “I work under a great deal of tension”, “I have felt fidgety or nervous as a result of my job” and “If I had a different job, my health would probably improve”. Work/life balance [71] was determined by a 4-item measure which required owner/mangers to indicate their level of agreement, time on a 7-point scale (strongly disagree, disagree, somewhat disagree, neither agree nor disagree, somewhat agree, agree, strongly agree), to statements such as “I currently have a good balance between the time I spend at work and the time I have available for non-work activities” and “I have difficulty balancing my work and non-work activities”. 

#### 2.2.7. Health-Related Factors

Health-related factors chosen for exploration were psychological treatment and self-rated health. We have previously reported that receiving treatment for depression was negatively correlated with presenteeism and having excellent, very good or good self-rated health was positively correlated with presenteeism [51]. Further, we suggest this finding may indicate that individuals reporting presenteeism may be the less disabling cases of depression. Similar findings are expected in the SME setting [51].

A single item measure, the first item of the SF-12, assessed general self-rated health. This is a general indicator of self-reported health [72], which has been validated as a measure of general health status in various populations [73,74]. Self-rated health is related to important health outcomes including health risk behaviours, disability and mortality [75], and demonstrates good reliability and reproducibility [75]. Treatment was measured by participant responses regarding the receipt of professional medical help for a mental health concern in the three months prior to the survey. Specifically, responses to the item “Have you sought help from a professional in the past three months for a mental health concern”. If owner/managers answered affirmatively they were asked to specify whether that professional was a general practitioner, psychologist, or other type of health professional. 

### 2.3. Statistical Methods

Once the proportion of SME owner/managers reporting low/moderate and high/very high psychological distress was identified (Aim 1), the mean past–month sickness absenteeism days and presenteeism days and related standard deviations were calculated (Aim 2) (Table 2). Average self-reported lost productive time, expressed as the percentage of time SME owner/managers thought they were as productive as usual when they continued to work whilst ill, was also calculated for owner/managers reporting low/moderate and high/very high psychological distress (Aim 2).

Negative binomial regression was used to demonstrate the independent effects of socio-demographic, work-related wellbeing and health-related factors, as well as various individual and business characteristics on continuous measures of absenteeism and presenteeism days (Aim 3). This analysis was chosen as absenteeism and presenteeism days were both count variables with skewed distributions (Table 3). This method has been used in previous studies exploring the correlates of these work attendance behaviours [31].

## 3. Results and Discussion

Mean absenteeism and presenteeism days and the proportion of owner/managers reporting absenteeism and presenteeism (none *vs.* any) were calculated for all participating owner/managers, and by psychological distress category (low/moderate *vs.* high/very high) (Table 2). 

**Table 2:** Proportion of absenteeism and presenteeism days and mean absenteeism and presenteeism daysamongst SME owner/managers by psychological distress category.

	All Owner/Managers (*N =* 217)		Owner/Managers with Low/Moderate Psychological Distress (*N =* 137)		Owner/Managers with High/Very High Psychological Distress (*N =* 80)		
	*N*	%	*N*	%	*N*	%	*p*
Absenteeism							
Yes	58	26.7	27	19.7	31	38.8	0.002
No	159	73.3	110	80.3	49	61.2	
Presenteeism							
Yes	144	66.4	78	56.9	66	82.5	0.0001
No	73	33.6	59	43.1	14	17.5	
	Mean	S.D.					
Absenteeism Days	1.3	0.4	0.4	0.1	2.8	1.1	0.004
Presenteeism Days	6.2	0.5	3.2	0.5	11.2	1.0	<0.00001

**Figure 2:**
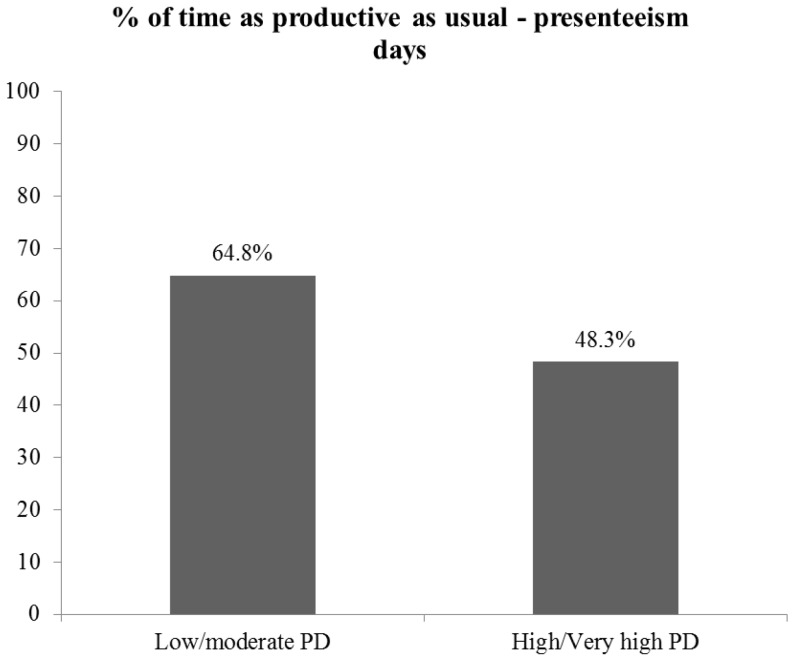
Inefficiency amongst SME owner/managers reporting past month presenteeism by psychological distress.

The mean percentage of self-reported inefficiency was calculated for owner/managers in both psychological distress categories (low/moderate *vs.* high/very high) reporting any presenteeism days (Figure 2).

### 3.1. Psychological Distress

Within our sample of 217 owner/managers, 63.1% (*N =* 137) met criteria for low/moderate psychological distress as measured by the K10 Psychological Distress Scale and almost 37% (*N =* 80) met criteria for high/very high psychological distress (Hypothesis 1).

#### 3.1.1. Past Month Sickness Absenteeism and Presenteeism by Psychological Distress

Table 2 displays the prevalence of psychological distress by work attendance behaviours and reported inefficiency (Hypothesis 2). Firstly reported is the incidence of absenteeism and presenteeism, whether or not any absenteeism and presenteeism days were reported, followed by the mean number of absenteeism and presenteeism days for those owner/managers reporting low/moderate psychological distress (*N =* 137) compared to those reporting high/very high psychological distress (*N =* 80) as well as for the total sample (*N =* 217). 

Approximately 66% (*N =* 144) of SME owner/managers reported attending work when ill in the previous month and just over 26% (*N =* 58) reported past month absenteeism. SME owner/managers reporting high/very high psychological distress reported taking a sickness absence significantly more than those with low/moderate psychological distress (*p =* 0.002). SME owner/managers reporting high/very high psychological distress also reported significantly more past-month presenteeism (*N =* 66, 82.5%; *p =* 0.0001) than those with low or moderate psychological distress. Owner/managers reporting high/very psychological distress also reported, on average, significantly more absenteeism (*p =* 0.004) and presenteeism days (*p <* 0.00001) than those reporting low/moderate psychological distress.

#### 3.1.2. Total Absenteeism and Presenteeism by Psychological Distress

Negative binomial regression models were used to determine the correlates of total absenteeism and presenteeism days (Table 3). 

**Table 3:** Correlates of absenteeism and presenteeism for SME owner/managers reporting high/very high psychological distress.

Variable	Total Absenteeism Days	Total Presenteeism Days
	β	β
Age	−0.09	−0.07
Gender	0.99 *	0.26
Education	1.29 **	0.27
Employees supervised	0.03	−0.12
Firm size	0.20	−0.11
Work hours	−0.13	−0.35
Treatment	1.42 ***	0.57 **
Self-rated health	0.29	0.77 **
Business confidence	0.03	−0.09
Job tension	−1.01 *	0.35
Job satisfaction	−1.81 **	−0.46
Work/life balance	0.86	−0.23
Conscientiousness	−0.73	−0.23
Neuroticism	1.54 ***	0.14

* *p* ≤ *0.1*; ** *p* ≤ 0.05; *** *p* ≤ 0.01; *Note*: Total absenteeism and presenteeism days analysed with negative binomial regression.

#### 3.1.3. Correlates of Absenteeism and Presenteeism

Absenteeism Days: Education (β = 1.29, *p =* 0.05), treatment (β = 1.42, *p =* 0.009), and neuroticism (β = 1.54, *p =* 0.01) were positively associated with past–month absenteeism days. That is, SME owner manager with a post high school education reported more past-month absenteeism days, as did those in who reported receiving treatment for a mental health issue in the past 3 months, and those with high neuroticism. Job satisfaction (β = −1.81, *p =* 0.01) was negatively associated with past-month absenteeism days. That is, SME owner/managers with high job satisfaction reported less past-month absenteeism days. Non-significant trends were also identified; job tension (β = −0.63, *p =* 0.084) was negatively associated with absenteeism and gender (β = 0.99, *p =* 0.07) was positively associated with absenteeism. Female SME owner/managers were more likely to report absenteeism than men and SME owner/managers reporting high job tension reported less past-month absenteeism days. 

Presenteeism Days: Analyses revealed that treatment (β = 0.57, *p =* 0.04) and self-rated health (β = 0.77, *p <* 0.0001) were positively associated with total presenteeism days. Owner/managers with excellent, very good or good self-rated health reported almost 12 times the average number of presenteeism days than owner/managers with fair or poor self-rated health. 

#### 3.1.4. Inefficiency due to Presenteeism by Psychological Distress

SME owner/managers reporting presenteeism days were also asked to report their degree of inefficiency, or productivity loss, on the days they attended work when ill. Figure 2 represents the mean inefficiency for the total sample by K10 Psychological Distress category. Reported inefficiency varied considerably between those owner/mangers in the low/moderate psychological distress category as compared to those owner/managers in the high/very high psychological distress category; that is substantial productivity loss occurs amongst owner/managers reporting very high psychological distress.

### 3.2. Discussion

The lack of current SME-focused mental health promotion strategies may be due to the dearth of research identifying which, if any, SME-specific factors prompt psychological distress and depression, the related work attendance behaviours, and associated health and economic consequences. This study attempted to address this research gap. It used self-report data from a sample of SME owner/managers to identify the proportion reporting high psychological distress, the number of past-month absenteeism and presenteeism days reported, the degree of inefficiency associated with continued work attendance, and which socio-demographic, work-related wellbeing, and health-related factors, as well as individual and business characteristics, that were associated with absenteeism and presenteeism days. 

Results revealed 36.9% (*N =* 80) of participating owner/managers reported high/very high psychological distress. This proportion is higher than that previously observed in the general working population. Further, 38.8% (*N =* 31) of whom reported past month absenteeism and the majority reported attending work when ill (*N =* 66, 82.5%). This finding may due to magnified work attendance pressures experienced by SME owner/managers. Further research is needed to determine whether these findings are due to pressures associated of owning and managing an SME and/or the lack of information, support and time available to manage these pressures. 

Additionally, owner/managers reporting high/very high psychological distress and presenteeism presenteeism estimated they were as productive as usual less than 50% of the time. As the exploration of the potential correlates of absenteeism and presenteeism amongst owner/managers reporting high/very high psychological distress revealed no SME-specific factors, the identification of this substantial presenteeism-related inefficiency is arguably this study’s most important finding. The potential threat this lost productive time could pose to the continued sustainability and profitability of small-to-medium businesses highlights the findings economic significance and demonstrates the importance of managing poor mental health and related work attendance behaviours in SMEs.

Although this study failed to identify any SME specific factors correlated with presenteeism, it did identify an association between receiving treatment and work attendance that was at odds with the findings from previous investigations of presenteeism correlates. We have previously found that within a population of employed Australian adults reporting lifetime major depression, treatment was negatively associated with presenteeism [51]. Further, as absenteeism was measured as the converse of presenteeism, treatment was positively associated with taking a sickness absence. However, in our sample SME owner/managers who reported receiving treatment from a GP, psychologist or other health professional for a mental health issue in the 3 months prior to the survey reported more absenteeism and presenteeism days than those who had not sought treatment during that period. 

We have previously suggested the association with absenteeism may be due individuals receiving treatment experiencing more severe symptoms, which require them to seek treatment and compel their absence from work [51]. By extension they suggest presenteeism reporters are the milder cases of depression [51]. Although treatment is positively correlated with presenteeism in this study the relationship was not as strong as that between treatment and absenteeism, suggesting the aforementioned explanation may still be applicable. Using our understanding of the work attendance pressures SME owner/mangers experience, we could hypothesise that SME owner/managers receiving treatment are in fact the more severe cases of depression but due to various individual and organisational features, which compel their presence they continue to work, whereas the same circumstances in a larger organisation may allow absence. Therefore, special attention should be paid to owner/managers reporting high/very psychological distress as they are likely to continue working which may have potentially significant consequences in terms of the long term health of the individual, as well as significantly impact the profitability and sustainability of their business due to their reduced capacity to work productively as demonstrated by the findings of this study. 

That said, it must be considered that the differences between our previous findings [51] and those of the present study may be explained by the difference in the way presenteeism was operationalized. Specifically, our previous study [51] defined presenteeism as the absence of absenteeism and therefore absenteeism and presenteeism were mutually exclusive outcomes categories. That is, respondents could be in one or the other, not both. Further, absenteeism and presenteeism reported was in reference to the 12 months prior to the survey interview. However, the SME owner/mangers in this study were asked two separate questions about whether they experienced past-month absenteeism and past-month presenteeism and could therefore report experiencing both absenteeism and presenteeism. 

The positive correlation identified between better self-rated health and presenteeism days more convincingly supports the suggestion that individuals who continue to work whilst experiencing high psychological distress are experiencing less disabling symptoms, and thus more able to work. Despite reporting high/very high psychological distress, rating their health as favourable was a more powerful correlate of continued work attendance for participating owner/mangers. This is consistent with previous findings which suggest an individual’s decision to continue working is strongly conditional on their self-assessed health level [76]. This is likely to be the case for SME owner/managers who, due to features such as small work teams and multiple role responsibilities, are unable to rely on colleagues or employees to compensate for the lost productivity their sickness absence would yield. However, despite considering their self-rated health adequate enough to rule out taking a sickness absence those owner/managers who continued to work whilst experiencing high/very high psychological distress reported substantially reduced productivity. Consequently, ratings of psychological distress should be considered before self-rated health scores when identifying which owner/managers should be the initial targets for future workplace mental health promotion strategies and interventions. Alternatively, this finding may also suggest incorporating mental health into general health promotion strategies could be effective as physical health may buffer the relationship between psychological distress and work attendance.

#### 3.2.1. Limitations

Research trials often yield samples unrepresentative of the general population and the baseline data analysed here revealed this sample is no exception (Table 1). For example, we have a higher proportion of women than in the broader SME population. This may be explained by the consistent research finding that women experiencing depression are more likely to disclose symptoms and seek treatment [77,78,79], or in the case of this study, enter an intervention trial evaluating a workplace mental health promotion tool. This elucidation is supported by the findings of a recent, national survey of mental disorder prevalence which reported approximately 40% of women with a mental disorder reported service use compared to 28% of men [80]. 

The distribution of participating owner/managers by industry also differed from the most recently released nationally representative SME information (Table 1). For example, almost 16% of our sample works in the health industry (Table 1), which could suggest they are more health, and mental health, literate. Health literacy refers to is the knowledge and skills needed to understand and use information relating to health issues and staying healthy [81]. Mental health literacy has been defined as “knowledge and beliefs about mental disorders which aid their recognition, management or prevention” and includes the ability to recognise specific disorders, knowing how to seek mental health information, knowledge of risk factors and causes, and professional help available, and attitudes that promote recognition and appropriate help-seeking [82]. Such skills and knowledge of mental health may serve to differentiate the SME owner/managers in this study from the broader SME population who may not be as able to recognise, manage, seek help for or prevent mental health difficulties. Further, the mental health literacy of the participating owner/managers may have prompted their decision to participate in the intervention program, meaning there are likely to be SME owner/managers who aren’t health literate and therefore did not engage with the program, for whom the stress involved in running a small business, and the subsequent psychological distress, work attendance behaviours and lost productive time are even more pronounced. 

The representativeness of the sample used in this study may have also been compromised during the recruitment and registration stages of the program. A large proportion of owner/managers who registered to participate reported doing so as they had experienced mental health difficulties, or their staff had. This may have produced an over-estimate of psychological distress within our sample and the findings cannot be considered representative of SME owner/managers beyond those who volunteered to participate in our mental health promotion intervention. However, as this is the first research of this type to be conducted in a SME setting it is an important starting point and indicates the need for more research to be conducted with a more representative sample.

Therefore, the sample analyses in this study are not representative of the broader small business community. Subsequently, the prevalence estimates are unable to be generalised to SMEs nationwide. Further, as the research was only conducted amongst SME owner/managers, conclusions cannot be drawn about employees within this sector, who make up the majority of the private sector workforce in most developed economies worldwide. Additionally, the cross sectional design of the survey meant causal inferences cannot be made regarding the association between the reported factors and absenteeism and presenteeism behavior in this population. Future research should aim for a systematic sampling procedure in order to achieve a representative sample and remove these aforementioned biases.

Another potential limitation of this study, which was dictated by the small sample size, is the aggregation of SME owners and managers into one group. That is, differences between owners and managers in terms of personality traits or characteristics and their level of personal and financial involvement in the business have the potential to affect their probability of experiencing high/very high psychological distress and influence their work attendance decisions. For example, a manager of a SME may experience a sense of belonging and personal commitment to the business, driven by the small work terms and a close working relationship with the business owner, which may compel presenteeism. However, owners may be even more motivated to continue working when sick in order to prevent lost productive time due to sickness absence as they also have a financial stake in the continued profitability and sustainability of the business. That said, 68% of SME owner/managers in this study identified themselves the business owner, CEO or director. The remainder were senior managers, who reported a high level of responsibility regarding the day-to-day running of the business, and subject to many of the same operational, workplace stresses as the SME owners. Therefore, aggregation is unlikely to influence the results of this study. 

The small sample size also prevented the construction of a more complex multivariable model, accounting for potential confounders, in order to identify which factors were associated absenteeism and presenteeism within this population. That said, identifying the work and non-work factors associated with work attendance behaviour amongst SME owner/managers reporting high/very high psychological distress was only one part of what this study was attempting to address. Arguably more noteworthy is the identification of high rates of psychological distress within our sample of SME owner/managers and the lost productive time or inefficiency associated with the related absenteeism and presenteeism behaviours amongst those reporting high or very high psychological distress. These findings provide evidence of the pressing need to better manager psychological distress and related sickness absence and presenteeism within this sector.

#### 3.2.2. Strengths

The strength of this study lies in providing the first estimates of absenteeism and presenteeism amongst SME owner/managers reporting high/very high psychological distress, the related inefficiency or lost productive time, and which work- and non-work-related variables are associated with these work attendance. To our knowledge no other study has attempted to address this within the SME sector despite the potentially costly health and economic implications, and doing so represents an important contribution to occupational health literature which has, to date, neglected the SME sector despite the sizeable contribution they make to most developed economies worldwide. 

## 4. Conclusions

This study revealed the proportion of SME owner/managers reporting high/very high psychological distress and the substantial lost productive time reported by owner/managers reporting related presenteeism. Such findings highlight the value of the Business in Mind program, by confirming the need for workplace mental health promotion programs developed to reduce the incidence of sickness absence and continued work attendance, the number of absenteeism and presenteeism days reported, and the associated productivity loss in SMEs. This proposition is supported by a recent systematic review of the effect of workplace health promotion programs on reducing presenteeism which revealed involving supervisors and managers and improving supervisor/manager knowledge of mental health were common attributes of successful programs [83]. Therefore, workplace mental health promotion programs tailored to the SME sector, and designed to improve owner/managers awareness of mental health issues, amongst themselves and their employees, are to be encouraged. 

Before this can be achieved, more research is needed, ideally using a larger sample and longitudinal data to identify which factors cause or prompt absenteeism and presenteesim amongst SME owner/mangers reporting high/very high psychological distress. Further, follow up data is required to provide longitudinal evidence of the effectiveness of workplace mental health promotion programs focused on SMEs, such as the Business in Mind program. This information could inform more targeted workplace health promotion interventions in this sector and potentially increase their chances of success. Such measures have the potential to benefit employees, employers and economies worldwide through investment in the health and productivity of the SME workforce.
